# Biomaterial-Based and Surgical Approaches to Local Hemostasis in Contemporary Oral Surgery: A Narrative Review

**DOI:** 10.3390/jfb16050190

**Published:** 2025-05-21

**Authors:** Atanaska Dinkova, Petko Petrov, Dobromira Shopova, Hristo Daskalov, Stanislava Harizanova

**Affiliations:** 1 Department of Dental, Oral, and Maxillofacial Surgery, Faculty of Dental Medicine, Medical University-Plovdiv, 4000 Plovdiv, Bulgaria; hristo.daskalov@mu-plovdiv.bg; 2 Department of Maxillofacial Surgery, Faculty of Dental Medicine, Medical University-Plovdiv, 4000 Plovdiv, Bulgaria; petko.petrov@mu-plovdiv.bg; 3 Department of Prosthetic Dentistry, Faculty of Dental Medicine, Medical University-Plovdiv, 4000 Plovdiv, Bulgaria; 4 Department of Hygiene and Ecomedicine, Faculty of Public Health, Medical University-Plovdiv, 4000 Plovdiv, Bulgaria; stanislava.harizanova@mu-plovdiv.bg

**Keywords:** local hemostasis, bleeding, oral surgery, tranexamic acid, gelaspon, fibrin sealants

## Abstract

Effective local hemostasis is essential in oral surgery to prevent complications such as delayed healing, infection, and the need for re-intervention. Postoperative bleeding occurs in 4–6% of cases, increasing to 9–12% in patients receiving anticoagulant or antiplatelet therapy. This review evaluates the efficacy, safety, and clinical utility of local hemostatic agents based on 51 studies published between 1990 and 2023. Traditional agents, such as oxidized cellulose and gelatin sponges, control bleeding in over 85% of standard cases but offer limited regenerative benefits. Autologous platelet concentrates (APCs), including platelet-rich plasma (PRP) and leukocyte- and platelet-rich fibrin (L-PRF), reduce bleeding time by 30–50% and enhance soft tissue healing. Studies show the PRP may reduce postoperative bleeding in dental surgery by 30–50%, and in orthopedic and cardiac surgery by 10–30%, particularly in patients on anticoagulants. Tranexamic Acid mouthwash can reduce postoperative bleeding by up to 50–60%. Fibrin sealants achieve a 70–90% reduction in bleeding among high-risk patients, while topical tranexamic acid decreases hemorrhagic events by up to 80% in anticoagulated individuals without increasing thromboembolic risk. However, comparative studies remain limited, particularly in medically compromised populations. Additional gaps persist regarding long-term outcomes, cost-effectiveness, and the standardized use of emerging agents such as nanomaterials. Future research should prioritize high-quality trials across diverse patient groups and develop clinical guidelines that integrate both safety and regenerative outcomes.

## 1. Introduction

Post-operative hemorrhage is a significant concern in surgical procedures, as it can lead to severe complications and adversely affect patient outcomes [1,2]. Bleeding following surgery may result from inadequate intra-operative hemostasis, underlying coagulation disorders, or the resumption of anticoagulant therapy in the post-operative period. In oral and maxillofacial surgeries, such as tooth extractions or implant placements, post-operative bleeding can increase patient morbidity, delay wound healing, and elevate the risk of infection. Therefore, prompt and effective local hemostasis is essential to minimize these risks and ensure optimal surgical outcomes [3,4].

Local hemostatic agents play a particularly important role in managing post-operative bleeding when systemic anticoagulation cannot be interrupted, or in high-risk patients, such as those with cardiovascular diseases, who cannot safely discontinue their medication [5,6,7]. These agents enable localized control of bleeding without affecting systemic coagulation, offering a safer alternative to modifying systemic therapy. Recent studies have demonstrated the efficacy of local hemostatic agents—including collagen sponges, fibrin sealants, oxidized cellulose, and topical thrombin—in achieving targeted bleeding control, thereby reducing the risk of prolonged hemorrhage and related complications [8,9,10].

Hemorrhage is an unpleasant, unwanted, and dangerous complication of dental procedures. The proper selection and use of local hemostatic agents are essential for minimizing complications, promoting healing, and ensuring patient safety, highlighting their importance in contemporary surgical practice. This article reviews current local hemostatic techniques and offers evidence-based guidance to support dentists in making informed clinical decisions.

## 2. Materials and Methods

A narrative literature search was conducted using Medscape, PubMed-MEDLINE, ScienceDirect, and EBSCOhost to ensure a broad and comprehensive collection of peer-reviewed articles published between 1990 and 2023. The keywords included “local hemostasis”, “post-operative bleeding”, “oral surgery”, “tranexamic acid”, “gelaspon”, “fibrin sealants”, “collagen sponges”, “oxidized cellulose”, “topical thrombin”, “autologous platelet concentrates (APCs)”, and “PRP (platelet-rich plasma)”. The inclusion of APCs and PRP was essential to reflect the growing interest in biological agents that promote both hemostasis and tissue regeneration.

Following an initial screening of 548 publications, 51 studies were identified as highly relevant to local aemostasis in oral surgery. Priority was given to studies evaluating the effectiveness, safety, and clinical applications of local hemostatic agents.

Data Extraction and Selection Process:

Inclusion criteria: full-text articles written in English; review articles and original research related to local hemostasis during dental extraction.

Exclusion criteria: non-English articles, case reports, editorials, commentaries, letters, and abstracts were excluded due to the lack of rigorous data. Studies not related to local hemostasis in oral surgery, focusing on non-surgical treatments, or lacking outcomes such as bleeding reduction, hemostasis time, adverse reactions, or clinical utility were also excluded.

Study Selection:

Two independent researchers reviewed the articles. Studies were included if they provided quantitative or qualitative data on the efficacy, safety, or clinical use of local hemostatic agents in oral surgery.

Categorization of Agents:

Selected studies were categorized by agent type:

- Traditional agents: e.g., tranexamic acid, gelaspon, fibrin sealants;
- Mechanical agents: e.g., collagen sponges, oxidized cellulose;
- Topical agents: e.g., topical thrombin;
- Biological agents: e.g., autologous platelet concentrates (APCs), PRP, L-PRF.

Key Data Extracted:

- Efficacy (e.g., bleeding reduction, hemostasis time);
- Safety (e.g., adverse reactions, infection risk);
- Clinical utility and comparative performance.

**Figure 1:**
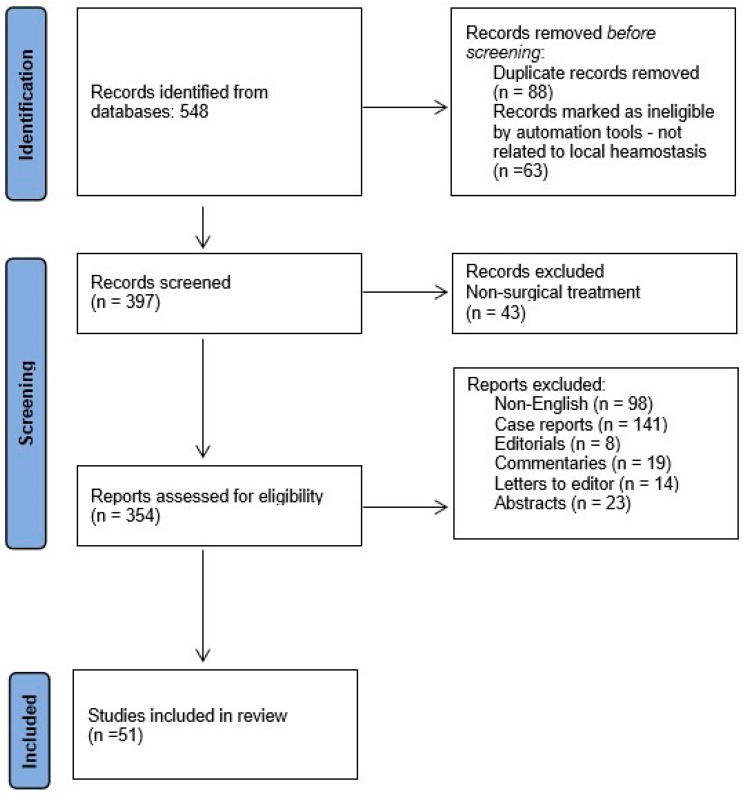
Flowchart of data collection.

The article selection process is illustrated in Figure 1.

## 3. Narrative Review

Bleeding during and following dental procedures may arise from a range of risk factors, broadly classified as systemic (general) and local. A thorough understanding of these etiological factors is critical for the appropriate selection and application of hemostatic agents and techniques to ensure effective bleeding control. The primary risk factors contributing to bleeding include general factors [11,12,13,14,15,16] and local factors [17,18,19,20,21]. They are presented in Table 1.

Local hemostatic agents are a primary tool for dentists to achieve effective hemostasis following dental surgery, particularly in patients with impaired coagulation.

A study by Takahara et al. [11] demonstrated that local hemostatic agents significantly reduced postoperative bleeding complications in patients undergoing oral surgery while receiving anticoagulant therapy. Similarly, a systematic review by Coelho et al. [12] reported that the use of agents such as tranexamic acid mouthwash, in combination with physical barriers like gauze, effectively minimized postoperative bleeding, enabling the safe continuation of anticoagulant treatment. Furthermore, a review by Karsli et al. [13] emphasized that local hemostatic techniques, when combined with meticulous surgical protocols and appropriate patient monitoring, are critical for improving outcomes in anticoagulated patients undergoing both oral and general surgical procedures. Peck and Anderson [14] examined the efficacy of autologous platelet concentrates in oral surgery, highlighting their dual role in promoting hemostasis and enhancing tissue healing.

Numerous other clinical studies further support the effectiveness of local hemostatic agents in achieving reliable hemostasis in patients with compromised coagulation profiles [19,21,22].

The local hemostatic methods can be classified as in Table 2:

1. **10–20% Sodium Chloride solution** (Saline)

A hypertonic solution of sodium chloride (saline) has long been used as a topical hemostatic agent. When applied with mechanical compression using sterile gauze, it exerts its hemostatic effect through several mechanisms. The high osmolarity draws tissue fluid into the blood vessels, promoting the release and transport of tissue thromboplastin, which initiates the extrinsic coagulation pathway [7]. Additionally, the hypertonic environment induces mild hemolysis of erythrocytes and platelets, resulting in the release of thromboplastic substances that further amplify the coagulation cascade by enhancing the activity of endogenous thromboplastin [19].

This method is particularly useful in controlling minor external bleeding, especially in settings such as dental surgery, ENT procedures, or superficial skin injuries. Its localized action, ease of use, and low cost make it a practical adjunct for temporary hemostasis when other agents are unavailable or unsuitable [17].

2. **Hydrogen Peroxide solution 3–6%**

Hydrogen peroxide acts as a topical hemostatic agent primarily through its vasoconstrictive and pro-coagulant effects. Its mechanism of action is promoting vasoconstriction by increasing intracellular calcium in smooth muscle cells, stimulating the formation of cyclooxygenase-derived prostanoids, activating various enzymes and potassium channels, and generating hydroxyl radicals, which contribute to vessel constriction and clot stabilization [23].

Hydrogen peroxide solution **is** commonly used in dental surgery and minor procedures. It is applied topically with sterile gauze to control bleeding, clean wounds, and reduce bacterial load, making it a practical agent for managing superficial bleeding and wound care [4,13].

3. **Aprotinin** (Trasylol-Fresenius Kabi Austria GmbH Graz, Austria; Gordox-Gedeon Richter, Budapest, Hungary)

Aprotinin is a polyvalent protease inhibitor with potent antifibrinolytic activity. It works by inactivating enzymes such as plasmin, trypsin, alpha-chymotrypsin, and kininogenases, thereby preventing fibrin degradation and stabilizing blood clots [24,25].

Aprotinin inhibits plasmin and other proteolytic enzymes involved in fibrinolysis, reducing clot breakdown and promoting hemostasis.

In dental practice, aprotinin (100,000 KUI) can be applied locally using sterile gauze to control bleeding during or after procedures. It is particularly useful in patients with bleeding tendencies or undergoing invasive dental surgeries.

Possible side effects include allergic reactions, hypotension, tachycardia, thrombophlebitis at the injection site, myalgia, bronchospasm, hallucinations, and cognitive disturbances.

Contraindications exist in individuals with known hypersensitivity to aprotinin [17].

4. **Adrenaline** (Par Sterile Products LLC, Spring Valley, NY 10977, USA)

Adrenaline (epinephrine) at a 0.1% concentration is used for local hemostasis, typically applied with sterile gauze. Its alpha-adrenergic agonist activity causes vasoconstriction of blood vessels in the skin, mucous membranes, and abdominal organs [26].

Adrenaline stimulates alpha-1 adrenergic receptors, leading to vasoconstriction in peripheral tissues and enhanced local hemostasis.

It is widely used in dental procedures and minor surgeries to control bleeding, especially in highly vascular areas, often in combination with local anesthetics.

Adverse reactions may include palpitations, tachycardia, hypertension, anxiety, tremor, headache, and sweating, especially if absorbed systemically.

It should not be used in patients with severe hypertension, hyperthyroidism, coronary artery disease, or arrhythmias due to the risk of cardiovascular complications [4,11].

5. **Etamsylate** (Dicynone-OM Pharma SA, Meyrin, Switzerland)

Etamsylate is a non-hormonal hemostatic agent that reduces bleeding by strengthening capillary walls and improving platelet function. It enhances platelet adhesion and aggregation at the site of injury, promoting the formation of a stable clot [27,28].

The mechanism of action is to stabilize the endothelium of blood vessels and stimulate platelet adhesion and aggregation, aiding primary hemostasis without directly affecting coagulation factors.

Clinical application is in various surgical settings, including dental and ENT procedures, as well as in menorrhagia and capillary bleeding, particularly when platelet function needs support.

It is not recommended for patients with hypersensitivity to the drug or with a history of thromboembolic disorders or active arterial thrombosis [4,11].

6. **Aminocaproic acid**

Aminocaproic acid is a synthetic antifibrinolytic agent structurally related to lysine. It works by competitively inhibiting plasmin and plasminogen activators, preventing fibrin degradation and stabilizing blood clots [29].

By mimicking lysine, aminocaproic acid blocks the binding sites of plasminogen and plasmin, thereby inhibiting fibrinolysis and preserving existing clots.

Clinically, it can be used in cases of bleeding due to fibrinolytic therapy, as an adjunct treatment in hemophilia, and topically in dental procedures to support clot stability.

Adverse effects can be hypotension, gastrointestinal discomfort, diarrhoea, nasal congestion, and intravascular thrombosis.

It is contraindicated in patients with disseminated intravascular coagulation (DIC) unless DIC is under control [18,29].

7. **Epsylone** (Aminocaproic Acid, EAC-Pfizer Inc., New York, USA)

Epsylone (aminocaproic acid) reduces fibrinolysin activity and interferes with the degradation of fibrin, thereby stabilizing clots and preventing excessive bleeding [29].

EAC works by inhibiting plasminogen activation, which reduces fibrinolysin activity, preventing the breakdown of fibrin and helping to maintain clot stability.

Epsylone can be used in a 15% gargling solution to control local bleeding, particularly in dental procedures or following oral surgery, where it helps reduce post-operative bleeding by stabilizing blood clots in the oral cavity [18,29].

8. **Paraaminomethylbenzoic acid** (PAMBA) (Takeda Pharmaceutical Company Limited, Konstanz, Baden-Württemberg, Germany)

Pambenzacid, containing paraaminomethylbenzoic acid, is an antifibrinolytic agent that works by inhibiting factors involved in blood clotting, such as various kinases, thus preventing the breakdown of blood clots [30,31].

Pambenzacid suppresses the action of kinases that are responsible for fibrinolysis, thereby reducing clot degradation and promoting hemostasis.

Pambenzacid is used to manage local bleeding due to increased fibrinolysis, such as after tonsillectomy, dental procedures, adenoidectomy, and in patients with hemophilia A and B, von Willebrand disease, or von Willebrand–Jürgens syndrome. It is also used as an antidote in cases of anticoagulant overdose or fibrinolytic overdose, helping to counteract excessive bleeding from these treatments [30,31].

9. **Gelatin**

Gelatin is a hydrocolloid derived from purified animal collagen, available in forms such as a gelatin sponge, powder (to be mixed into a paste), or film. It can be used dry or moistened with a physiological solution [32,33,34,35].

When used as a hemostatic sponge, gelatin absorbs blood and platelets, facilitating the formation of active thromboplastin. This enhances clot formation, resulting in a massive clot that incorporates the gelatin sponge, helping to control bleeding.

Gelatin is widely used in dental procedures, surgical interventions, and trauma care. It can be used alone or combined with other hemostatic agents to enhance its efficacy in controlling bleeding, particularly in vascular surgeries or areas with extensive bleeding [11,33].

**9.1. Gelfoam^®^** (USP, Pharmacia & Upjohn Company LLC, 7000 Portage Road, Kalamazoo, MI, USA) is an absorbable gelatin powder from absorbable gelatin sponge. Gelfoam^®^ is derived from purified porcine (pig) skin gelatin. It has the ability to absorb many times its weight in whole blood. Gelfoam Sterile Powder, saturated with sterile sodium chloride solution, is indicated in surgical procedures, including those involving cancellous bone bleeding, as a hemostatic device, when control of capillary, venous, and arteriolar bleeding by pressure, ligature, and other conventional procedures is either ineffective or impractical. Although not necessary, Gelfoam can be used either with or without thrombin to obtain hemostasis. When not used in excessive amounts, Gelfoam is absorbed completely, with little tissue reaction within 4–6 weeks [36,37]. Mahmoudi et al. extracted two mandibular molar teeth in 26 patients and recorded the amount of bleeding for 1 and 4 h. They concluded that Gelfoam is recommended for use in dental surgeries because of its ability to control bleeding, pain, and dry sockets [38].

**9.2. Surgispon^®^** (Aegis Lifesciences Pvt. Ltd., Gujarat, India) is a resorbable, hemostatic gelatin sponge designed for hemostatic use by applying it to a bleeding surface. Surgispon^®^ is a hemostatic sponge manufactured from highly purified, premium-grade gelatin material for use in surgical procedures. When implanted in vivo in appropriate quantities, it is completely absorbed within 3–4 weeks. Applied to bleeding mucosal areas, it liquefies within 2 to 5 days. Surgispon^®^ gelatin sponges have a porous structure that activates platelets as soon as blood comes into contact with the sponge matrix, allowing them to act as a catalyst in fibrin formation. Surgispon^®^ can be effectively used for hemostasis in a variety of surgical procedures to control capillary, venous, or arterial bleeding by pressure. Surgispon^®^ should not be used in patients with known allergies to collagen, in combination with methyl methacrylate adhesives, in cases of blood leakage, for primary treatment of coagulation disorders, or in the presence of infection. When applied to mucosal surfaces, Surgispon^®^ can be left in place until it liquefies [21,33].

**9.3. Caprogel**^®^ (ISTA Pharmaceuticals, 15279 Alton Parkway, Suite 100 Irvine, CA 92618, USA) is a sterile resorbable hemostatic sponge containing medical gelatin, epsilon-aminocaproic acid, nitroxoline, and others. When applied locally to superficial wounds and during surgical interventions for parenchymal and capillary bleeding, the preparation stimulates blood clotting and stops bleeding. It is absorbed within approximately 30 days and does not cause allergic or toxic reactions. Caprogel^®^ is used as a local hemostatic agent for parenchymal and capillary bleeding during surgical interventions in neurosurgery, thoracic, abdominal, and maxillofacial surgery, gynecology, and urology [37].

**9.4. Gelaspon^®^** (Lauer FIS GmbH, Berlin, Germany) is a collagen-based hemostatic sponge used for controlling bleeding in surgery and medicine. This sponge is made from a large amount of collagen, which is a bioactive material known for its hemostatic properties. Gelaspon is applied as a compress or sponge on the bleeding surface, where it absorbs blood and stops bleeding by stimulating the coagulation process. After application, Gelaspon degrades in the tissues and is absorbed by the body relatively quickly [11,25]. Gelaspon has multiple medical applications, including in surgical procedures to control bleeding, wound healing, and other medical conditions where bleeding control is essential. A suitable-sized piece of the product can be cut with sterile scissors and placed on the bleeding site. The sponge can be applied dry or slightly moistened with sterile saline solution or freshly prepared thrombin solution in distilled water [30].

10. **Collagen Hemostatic Sponge**

**10.1. Tachocomb** (Nycomed (now part of Takeda Pharmaceutical Company Ltd., Zürich, Switzerland) contains collagen extracted from horse tendons and coated with human fibrinogen, bovine thrombin, and bovine aprotinin. Upon contact with bleeding tissues, coagulation factors in the product dissolve and, together with collagen, cover the lesions. Thrombin converts fibrinogen into fibrin, providing coverage of the wound surface along with polymerized collagen. Aprotinin prevents the plasmin-mediated lysis of the fibrin cover [22].

**10.2. Caprocol^®^** (Eucare Pharmaceuticals Pvt Ltd., Tamil Nadu, India) is a sterile resorbable sponge consisting of fibrillar collagen, epsilon-aminocaproic acid, nitroxoline, and excipients. It has a local hemostatic effect on superficial parenchymal and capillary bleeding. It is resorbed within 60 days without causing toxic or allergic reactions. It can be applied during surgical interventions for superficial parenchymal and capillary bleeding in neurosurgery, thoracic, abdominal, cardiovascular, maxillofacial surgery, gynecology, urology, and others [36].

11. **Surgicel** (Ethicon Inc., a subsidiary of Johnson & Johnson med tech, 1000 US Highway 202 S Raritan, NJ 08869, USA); **Oxysell** (Aegis Lifesciences, Gujarat, India)

Consists of an oxidized cellulose polymer (polyanhydroglucuronic acid), introduced into clinical practice in 1947, and is widely used in oral and maxillofacial surgery to control intraosseous arterial bleeding [33]. Halfpenny W et al. did a randomized trial with 46 patients taking warfarin with INR 2-4.1. For local hemostasis, they used oxycellulose (Surgicel) or fibrin glue (Beriplast). They reported 2 cases of bleeding after 24 h. They concluded that in patients with an INR within the therapeutic range, fibrin glue was as effective as oxycellulose. The single patient requiring hospitalization for hemorrhage had the most extracted teeth (six), the longest procedure (60 min), and was one of the oldest patients (81.8 years) [32].

Lillis T et al. published a study of 111 patients taking aspirin (42), clopidogrel (36), and aspirin and clopidogrel (42). They reported 66.7% bleeding in the first 30 min after extraction in patients with dual antiplatelet therapy, 2.6% with aspirin or clopidogrel monotherapy, and 0.4% in the control group, differences that were statistically significant. All complications were successfully managed with oxycellulose (Surgicel) and sutures. No patient developed late hemorrhage [39].

Morimoto Y. et al. extracted 513 teeth in 306 visits in 270 patients. All teeth were extracted without reducing the usual antithrombotic therapy. A total of 134 of the patients were taking warfarin, 49 were taking a combination of warfarin with an antiplatelet agent, and the remaining 87 were taking an antiplatelet agent alone. In patients on warfarin, the INR was 1.5–3.7. Oxidized cellulose and suture were used, and an appropriate local hemostatic effect was observed [22].

12. **Alveo Penga** (Produits Dentaires SA, Vevey, Switzerland)

A container containing 30 g of paste mixture of Penghawar Djambi contains the following: 4 g per 100 g, Iodoform: 10 g per 100 g, Butoform: 4 g per 100 g, Excipients q.s.p. 100 g. Alveo-Penga is a hemostatic agent that is applied through mechanical action; it facilitates clot formation (absorption properties of Penghawar Djambi) and also acts protectively at the operative site. Additionally, it contains Iodoform, which is an antiseptic whose action is particularly effective on mucous membranes and wounds. It helps prevent any possibility of infection, such as alveolitis. Butoform is a local contact anesthetic that reduces postoperative pain. It is used as a surgical hemostatic dressing after tooth extraction [40,41].

13. **Dry thrombin** (Thrombin-JMI^®^-Pfizer Inc., New York, NY, USA)

Thrombin is also known as activated Factor II in blood clotting. It converts fibrinogen into its active form, from which fibrin is synthesized, and it plays a significant role in platelet activation and the formation of a fibrin clot. It has an antagonistic effect against the action of vitamin K antagonists. It is obtained from human and bovine blood and is presented as a whitish powder in ampoules of 500–5000 IU. Before use, it is dissolved in saline solution/distilled water to a concentration of 100–1000 IU per cubic centimeter. The dissolved thrombin loses its activity after 24 h. Saturated gauze is applied to the bleeding area for 5–10 min [42].

Thrombin converts fibrinogen to fibrin, promoting clot formation and platelet aggregation, while counteracting vitamin K antagonists.

Clinically, it is used in surgical and dental procedures to control bleeding, for which thrombin is available as a powder (500–5000 IU) that is dissolved in saline or distilled water (100–1000 IU/cm^3^). Applied with saturated gauze for 5–10 min, it loses activity after 24 h [4].

14. **Fibrin Sealants** (Fibrin Glues)

Fibrin sealants, also known as fibrin glues, are hemostatic agents composed of fibrinogen and thrombin, which react together to form a fibrin clot upon application to tissues. They mimic the final stages of the blood coagulation cascade. Fibrin glue is applied topically to tissues during surgery. The components are typically stored separately and mixed just before application to ensure optimal activity. The mixture is applied directly to the surgical site, where it rapidly forms a fibrin clot, sealing tissues and promoting hemostasis. It is indicated in a variety of surgical procedures, including cardiovascular, orthopedic, general, and plastic surgery. It is particularly useful in operations involving tissues with a high risk of bleeding or requiring precise tissue adhesion. Monitoring for allergic reactions is recommended, although they are rare with fibrin sealants. [43,44].

Fibrin sealants mimic the final steps of the coagulation cascade, combining fibrinogen and thrombin to form a fibrin clot at the application site. When applied, fibrinogen is converted into fibrin, creating a biological adhesive that seals wounds, promotes hemostasis, and aids tissue repair.

Fibrin sealants are widely used in surgical procedures (e.g., cardiac, neurosurgery, and orthopedic surgeries) and dental procedures to control bleeding, seal anastomoses, and promote wound healing. They are especially useful in areas where sutures may be difficult or less effective, offering an effective hemostatic and adhesive solution. [17,44].

**14.1. Tissel** (Baxter Healthcare Corporation, Deerfield, MA, USA)

The fibrin sealant is a mixture of human fibrinogen and human thrombin solution, containing calcium chloride. This combination results in the activation of fibrinogen by thrombin, leading to the formation of a fibrin clot at the site of application. The addition of calcium chloride helps stabilize the fibrin clot formation by enhancing the enzymatic action of thrombin on fibrinogen. It is used in surgical procedures where rapid hemostasis and tissue sealing are required, particularly in cardiovascular, orthopedic, and general surgery. It is effective in controlling bleeding from small vessels and ensuring tissue adhesion and closure [45].

**14.2. Tissucol** (Baxter International Inc., Deerfield, MA, USA)

Tissucol is used as a fibrin sealant in surgical procedures to promote hemostasis and tissue sealing. It combines various clotting factors and proteins to mimic the final stages of the body’s clotting cascade, facilitating the formation of a fibrin clot to seal tissues. It is effective for controlling bleeding in cardiovascular, orthopedic, and general surgical procedures. Monitoring for signs of allergic reactions or adverse events during and after application is recommended. Tissucol acts by mimicking the natural clotting process, combining fibrinogen with thrombin and other clotting factors to form a stable fibrin clot at the site of application [46].

**14.3. Beriplast** (CSL Behring GmbH, Broadmeadows VIC 3047, Australia)

These kits contain human fibrinogen concentrate, aprotinin, human thrombin, and calcium chloride solution, components that are commonly used in surgical and hemostatic procedures to promote blood clotting and wound closure. The human fibrinogen and thrombin components work together to form fibrin, a crucial component of blood clots. Fibrin glues (Tissel, Beriplast) imitate the conversion of fibrinogen into fibrin, but due to a number of disadvantages (taking blood from the patient, allergic reactions, and high cost), they are not widely used in dental practice [46].

Al-Mubarak et al. [47] examined patients on anticoagulant therapy and concluded that they are safe and can be used in dental extraction at an INR of up to 5.0 and in surgical trauma in the range of 1 to 12. Similar results were published by other authors [48,49].

15. **Acidum tranexamicum** (Cyklokapron-Pfizer Inc., New York, NY, USA)

This mouthwash formulation of tranexamic acid is designed for the prevention and management of bleeding associated with fibrinolysis. It is administered through oral rinsing and is not intended for ingestion. The mouthwash protocol includes rinsing four times a day for 2–5 days. If bleeding persists, gauze soaked in the solution can be applied and pressed against the bleeding area. Due to its high cost, it is also not widely applicable in dental practice [50].

Tranexamic acid is a synthetic antifibrinolytic agent that works by inhibiting plasminogen activation, preventing the conversion of plasminogen into plasmin. Plasmin is responsible for the breakdown of fibrin in blood clots, so by blocking this process, tranexamic acid helps to stabilize clots and reduce bleeding.

Tranexamic acid is used in a variety of settings to manage excessive bleeding, including surgical procedures, trauma, heavy menstrual bleeding, and dental surgeries. It is particularly effective in treating bleeding disorders like hemophilia or post-operative bleeding. Tranexamic acid can be administered orally or intravenously, and it has a role in preventing bleeding during high-risk surgeries, such as cardiovascular or orthopedic surgeries [44,50].

Cardona-Tortajada et al. extracted 222 teeth in 155 patients taking antiplatelets, 26 of whom had minor bleeding successfully controlled with tranexamic acid [42].

**Figure 2:**
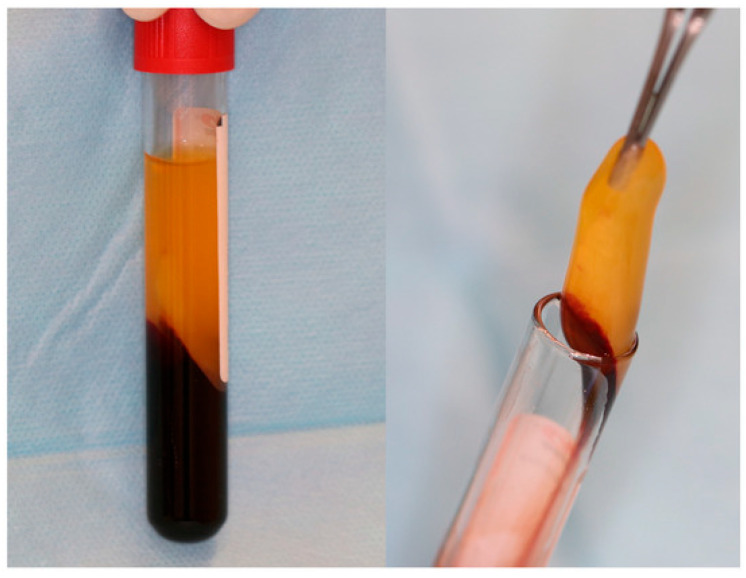
A-PRF clot in glass-coated plastic tubes. Reprinted from Ref. [51].

16. **Autologous platelet concentrates** (APCs), Figure 2, are biologically derived from a patient’s own blood, aimed at enhancing tissue healing and reducing inflammation. They contain a concentrated dose of platelets and growth factors that promote wound healing, tissue regeneration, and hemostasis [51].

The use of autologous blood products in surgery was first reported in 1954 by Kingsley, who used “platelet-rich human plasma” for its hemostatic and adhesive properties [45].

Autologous platelet concentrates (APCs) are blood products derived from the patient’s own blood, used to promote hemostasis and healing in surgical procedures. Research has shown that APCs contain a high concentration of platelets and growth factors, such as platelet-derived growth factor (PDGF) and transforming growth factor-beta (TGF-β), making them useful for managing post-operative bleeding and enhancing recovery in oral surgery.

APCs, such as platelet-rich plasma (PRP) and platelet-rich fibrin (PRF), contain a high concentration of platelets, growth factors, and fibrin matrix. Upon application, platelets release growth factors (e.g., PDGF, TGF-β, and VEGF) that promote tissue regeneration, enhance clot formation, and accelerate wound healing. The fibrin network provides a scaffold that supports cell migration and clot stability.

APCs are increasingly used as local hemostatic agents in oral surgery, implantology, periodontics, and maxillofacial procedures. They enhance hemostasis, reduce postoperative bleeding, and promote faster tissue repair and bone regeneration. Being autologous, they carry minimal risk of immune reaction or disease transmission. [4].

**Platelet-rich plasma (PRP)** contains a high concentration of platelets and growth factors such as platelet-derived growth factor (PDGF), transforming growth factor-beta (TGF-β), and vascular endothelial growth factor (VEGF) in a small plasma volume. It is widely used in oral surgery to minimize bleeding and promote tissue repair and regeneration [52].

Several studies have demonstrated the efficacy of APCs as hemostatic agents in oral surgery. Karsli et al. [13] highlighted that L-PRF not only provides effective hemostasis but also supports soft tissue healing through the release of growth factors. Another study by Bhandari et al. [53] reported that PRP significantly reduced bleeding times and improved recovery outcomes in patients undergoing tooth extractions compared to traditional hemostatic agents.

Leukocyte-rich platelet-rich fibrin (L-PRF) is a next-generation APC that includes platelets, leukocytes, and fibrin, which promotes rapid clot formation and tissue regeneration, making it highly effective in managing bleeding and improving wound healing in oral surgery [54].

Despite their promising benefits, the use of APCs in clinical practice is not without limitations. The preparation of APCs can be time-consuming and may require specialized equipment, which could hinder their use in routine dental practices. Additionally, the efficacy of APCs can vary based on the patient’s individual response, the preparation technique, and the specific surgical context. Some patients, particularly those with certain medical conditions (e.g., hematologic disorders), may not be suitable candidates for APCs, thus highlighting a gap in the applicability of these agents [55,56,57].

APCs (e.g., PRP and PRF) promote hemostasis and healing by delivering a high concentration of platelets and growth factors directly to the surgical site. These factors accelerate fibrin clot formation, angiogenesis, and tissue regeneration, while the fibrin matrix stabilizes the clot.

A comparative analysis with traditional hemostatic agents is presented in Table 3.

Despite the growing use of autologous platelet concentrates (APCs) like PRP and PRF, there is a lack of standardized protocols regarding their preparation, concentration levels, application methods, and clinical indications. This variability can lead to inconsistent outcomes, making it difficult to compare results across studies or replicate successful treatments reliably in clinical practice.

17. **Surgical suturing**

Atraumatic sutures are pre-threaded into a needle, minimizing tissue trauma and risk of infection and facilitating easier and quicker suturing. Surgical suturing is performed using surgical needles and sutures. Suture needles are available in a wide range, from small to large. This type of needle is supplied separately from the suture thread and has a hole through which the thread is passed. The advantage of this type of needle is that it allows for various combinations of needles and threads. However, eyed needles cause trauma to the tissues they pass through because the part with the eye is wider than the rest of the needle, and the double end of the thread causes additional tissue abrasion. Atraumatic needles do not have an eye, as the suture thread is factory-attached to the needle. The main advantage of this type of surgical needle is that there is no need to thread the needle, and tissue trauma during suturing is minimized due to the absence of an eye on the needle and the double end of the thread [58].

According to the curvature of the needle, the curved type can be in 1/2, 1,4, 3/8, 5/8, or in a J-shaped circle form. Surgical sutures, depending on the composition of the fibers, can be classified into monofilament and multifilament types. Monofilament sutures consist of a single filament, causing less tissue irritation when passing through, and they are less likely to serve as a reservoir for microbial growth [59].

Sutures can also be categorized based on their ability to be absorbed: absorbable and non-absorbable. During their absorption (taking 40 to 119 days), they do not provoke a tissue reaction, unlike silk sutures used in the past, which caused inflammation as a “foreign body”. Non-absorbable sutures are durable against proteolysis and hydrolysis. These sutures can sustain tension strength at 60 days. They should be removed 7–10 days after placement [60].

**Surgical sutures can be executed in various ways:**

**Figure 3:**
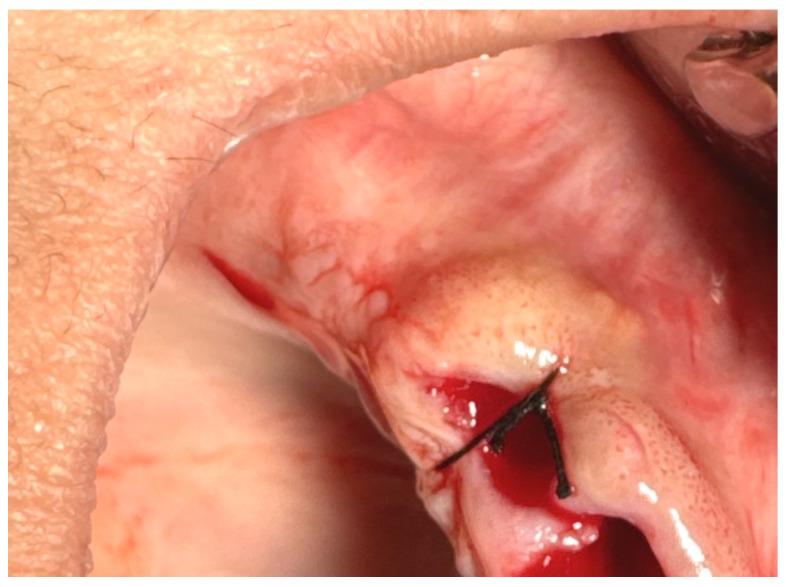
Single (vertical) interrupted suture. Reprinted with permission from Ref. [61]. Copyright 2024 Lax Book: Plovdiv.

**17.1. A single (vertical) interrupted suture** is the most common suture technique within the mouth cavity. The suture passes from one side of the wound, exits from the other side, and is knotted at the top, as shown in Figure 3 [59,60].

**Figure 4:**
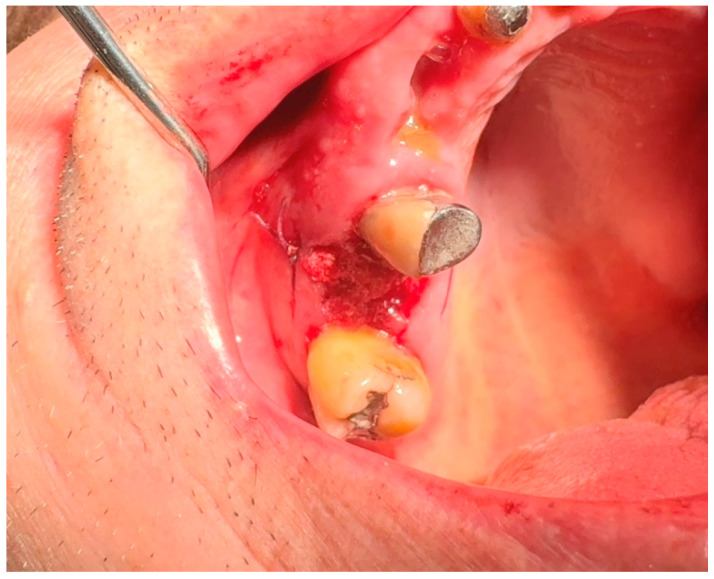
Horizontal U-shape (mattress) suture. Reprinted with permission from Ref. [61]. Copyright 2024 Lax Book: Plovdiv.

**17.2. A horizontal U-shape (mattress) suture** is used to assist in local hemostasis after tooth extraction, as shown in Figure 4. It tightens both sides of the wound (closing post-extraction wounds, covering intraosseous implants, etc.) [59,60].

**Figure 5:**
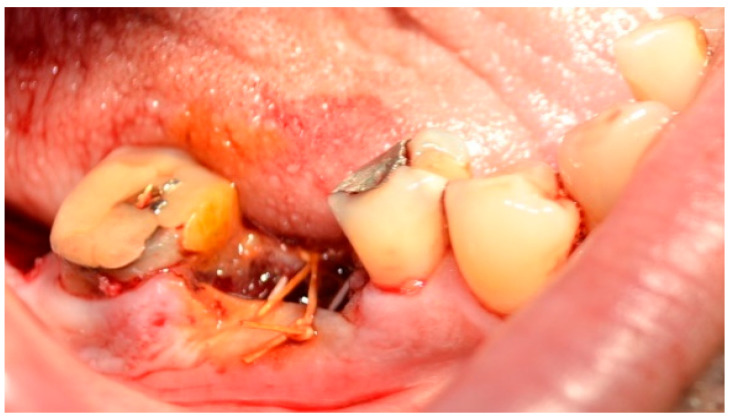
Horizontal U-shape locking suture. Reprinted with permission from Ref. [61]. Copyright 2024 Lax Book: Plovdiv.

**17.3. A horizontal U-shape locking suture** is used for additional tightening of the wound ends, ensures even tension distribution, prevents edge necrosis, and suits high-tension or mobile wounds, as shown in Figure 5 [59,60].

**Figure 6:**
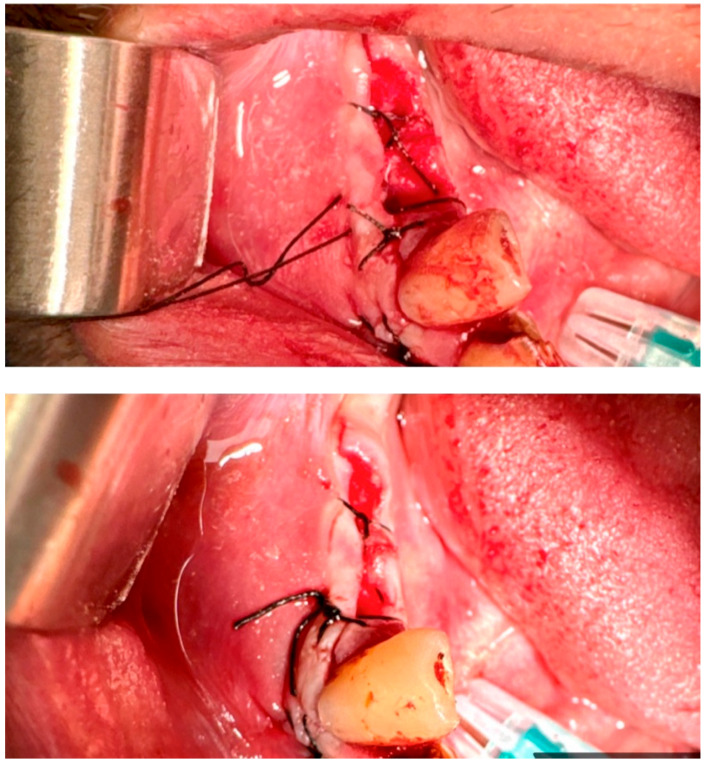
The figure-of-eight suture. Reprinted with permission from Ref. [61]. Copyright 2024 Lax Book: Plovdiv.

**17.4. Figure-of-eight suture**: This is a modified version of the horizontal mattress suture, as shown in Figure 6. While closing both sides of the soft tissue, figure-of-eight sutures help preserve the position of the clot [59,60].

**17.5. Vertical mattress suture (retentive suture):** This technique stabilizes wound edges and prevents closed space formation by passing the suture thread across the wound base. [48,49].

**CONTINUOUS SUTURES**

A continuous suture is used for long incisions, multiple extraction wounds, or wound closures that require uniform tension distribution. The suture is made with a continuous series of stitches without knotting between each stitch [48,49].

**Figure 7:**
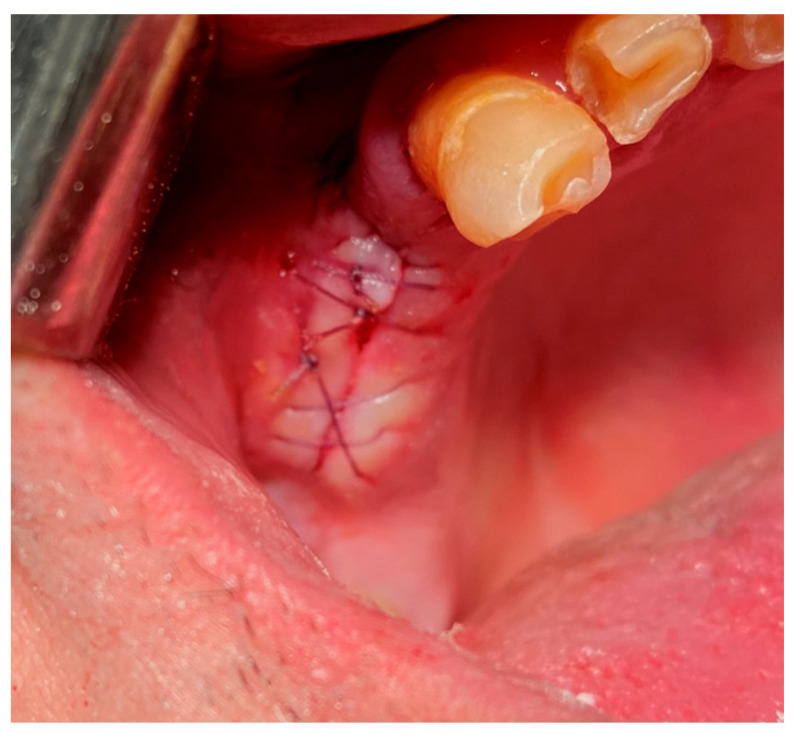
Simple continuous suture. Reprinted with permission from Ref. [61]. Copyright 2024 Lax Book: Plovdiv.

**17.6. Simple continuous suture:** In this technique, the suture material is run continuously along the wound edge, forming a series of closely spaced stitches. It is efficient for closing long wounds quickly, as shown in Figure 7 [59,60].

**Figure 8:**
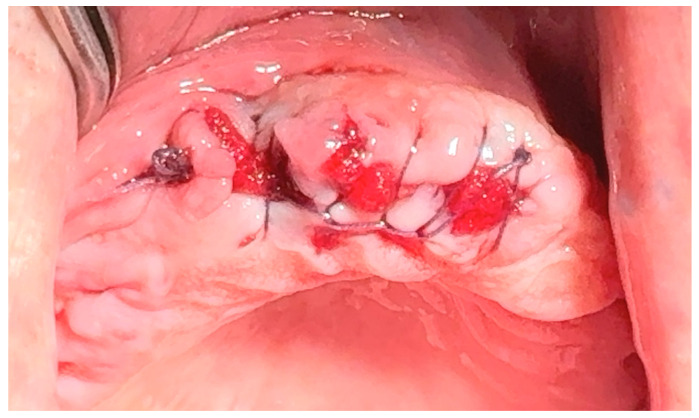
Simple continuous locking suture. Reprinted with permission from Ref. [61]. Copyright 2024 Lax Book: Plovdiv.

**17.7. Simple continuous locking suture:** This variation of the continuous suture includes additional locking stitches at specific intervals to enhance wound closure and stability, as shown in Figure 8 [59,60].

**17.8. Horizontal Mattress Suture:**

The horizontal mattress suture is used to evert wound edges and distribute tension evenly across the wound. It involves placing bites of tissue on both sides of the wound, resembling the shape of a “mattress” [59,60].

**17.9. Horizontal Mattress Locking Suture:**

**Figure 9:**
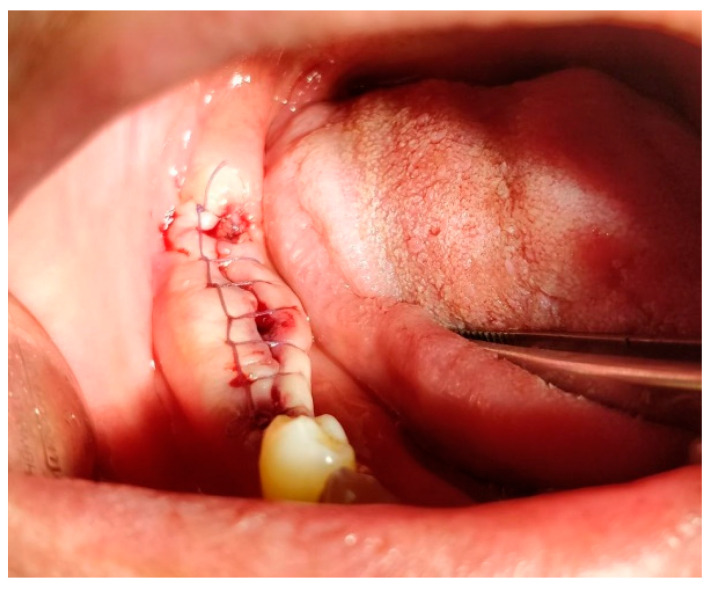
Horizontal mattress locking suture. Reprinted with permission from Ref. [61]. Copyright 2024 Lax Book: Plovdiv.

Similar to the horizontal mattress suture, this technique includes locking stitches to secure the wound edges more effectively [48,49]. Once placed, sutures are secured by tying knots manually using surgical instruments, as shown in Figure 9 [59,60].

Knots used in surgery can be simple (basic) or complex (multi-looped). Depending on the number of loops, knots can be as follows [62]:

- Single throw knot (one-loop knot): This is a simple knot with one loop around the suture material. It is quick to tie and provides basic security;
- Square knot (simple knot or granny knot): The square knot is a basic knot used in surgery, consisting of two throws or loops. It is commonly used for secure wound closure;
- Surgeon’s knot (two-handed knot, double throw knot): The surgeon’s knot is a more complex knot with additional throws or loops, designed to provide increased security and prevent loosening. It is commonly used in surgery to tie off sutures.

These knots are essential components of surgical suturing techniques and are selected based on the specific requirements of wound closure and tissue characteristics.

18. **Black tea**

In addition to the standard post-operative instructions following dental surgery, if bleeding resumes, patients are instructed to apply pressure to the wound with a black tea bag for 20 min. Black tea is rich in tannins, which have hemostatic, astringent, and mild antiseptic effects that help prevent infection at the extraction site [62].

## 4. Discussion

Local hemostatic agents play an essential role in oral surgical procedures, where prompt and effective bleeding control is fundamental for ensuring optimal wound healing and procedural success. While traditional hemostatic agents such as oxidized cellulose and gelatin sponges remain widely used due to their accessibility, ease of application, and predictable performance, advancements in biomaterials have introduced alternative agents with enhanced efficacy and regenerative potential [5,7,8,9].

**Traditional Hemostatic Agents**

Oxidized cellulose and gelatin sponges achieve hemostasis primarily through passive mechanisms; oxidized cellulose provides a matrix for platelet activation, while gelatin sponges absorb blood and induce clotting by volume expansion. These agents are generally effective for capillary or venous bleeding and remain foundational in clinical practice. However, their role is limited to achieving hemostasis and lacks contributions to active tissue regeneration, a critical limitation in complex surgical scenarios [17,18,22,23,27,29,35].

**Autologous Platelet Concentrates (APCs)**

Autologous platelet concentrates, including platelet-rich plasma (PRP) and leukocyte- and platelet-rich fibrin (L-PRF), have gained significant attention due to their ability to support both hemostasis and tissue regeneration. These products release a high concentration of growth factors, such as platelet-derived growth factor (PDGF), transforming growth factor-beta (TGF-β), and vascular endothelial growth factor (VEGF), that enhance angiogenesis, fibroblast activity, and collagen deposition. Clinical studies, such as those by Marenzi et al. [53,54], have demonstrated reduced bleeding time and improved soft tissue healing with PRP and L-PRF compared to traditional agents [14].

APCs are particularly advantageous due to their autologous origin, minimizing the risk of immunologic reactions and infection. Their regenerative benefits are especially relevant in procedures like periodontal therapy, implant placement, and ridge preservation [51]. However, their use is not without challenges. APC preparation requires centrifugation and trained personnel, and clinical efficacy may vary based on patient factors (e.g., baseline platelet count, systemic health). Furthermore, the lack of standardized preparation protocols leads to variability in outcomes across studies [52,55].

**Other Advanced Hemostatic Agents**

Other modern agents include fibrin sealants, microfibrillar collagen, and antifibrinolytics like tranexamic acid. Fibrin sealants closely mimic the final coagulation cascade and serve as both a hemostatic and regenerative scaffold. Tranexamic acid inhibits fibrinolysis and is particularly useful in patients with bleeding disorders or on anticoagulants. Clinical studies have shown that topical application of tranexamic acid can significantly reduce postoperative bleeding risks without increasing thromboembolic complications. However, its benefits are limited to clot stabilization and do not extend to tissue regeneration [29,43,48].

**Cost-effectiveness and accessibility**

Cost-effectiveness and accessibility are key considerations when selecting hemostatic biomaterials. Autologous platelet concentrates (APCs), such as PRF and PRP, offer a cost-effective option in practices equipped to prepare them, as they use the patient’s own blood and reduce the need for commercial products. However, they require specialized equipment and training, which may limit widespread accessibility [54,55,56,57]. Fibrin glues, while effective and easy to apply, tend to be more expensive and may not be readily available in all clinical settings, especially in resource-limited environments. Commercial hemostatic agents vary widely in price and effectiveness, underscoring the need to balance clinical benefit with economic feasibility when integrating these materials into routine care [43,44,45,46].

**Comparative Effectiveness and Evidence Limitations**

While various local hemostatic agents are available, direct comparisons between them are limited. Many existing studies differ in surgical context, evaluation methods, and outcome reporting, hindering comprehensive assessments of comparative efficacy. Moreover, while biologically active agents such as APCs show promise in enhancing soft tissue healing, their benefits in hard tissue regeneration are less well-defined [32,44,57].

**Research Gaps and Future Directions**

There is a critical need for high-quality, head-to-head comparative studies that evaluate local hemostatic agents across specific surgical indications. Future research should aim to standardize preparation and application protocols, especially for APCs, investigate combinations of agents to optimize both hemostatic and regenerative outcomes, and evaluate efficacy in medically compromised patients.

The main characteristics of hemostatic agents are summarized in Table 4.

## 5. Conclusions

While traditional hemostatic agents remain essential for effective bleeding control in oral surgery, autologous platelet concentrates (APCs) represent a significant advancement by combining hemostasis with enhanced regenerative capacity. Their application is especially valuable in complex or high-risk procedures where accelerated healing is critical. To fully realize their clinical potential, it is imperative to address the current lack of standardization and procedural consistency. Strategic integration of APCs, guided by evidence-based protocols and comparative clinical research, can transform perioperative care in oral and maxillofacial surgery, paving the way for more predictable outcomes and improved patient recovery.

**Future Directions:**

To strengthen clinical decision-making and expand the effective use of hemostatic agents in oral and maxillofacial surgery, future research should prioritize the following:

Randomized controlled clinical trials comparing autologous platelet concentrates (APCs) with traditional agents across various surgical procedures to evaluate efficacy, healing outcomes, and complication rates.

Cost-benefit analyses assessing the economic viability of APCs in routine and complex interventions, including long-term patient outcomes and healthcare resource utilization.

Standardization studies aimed at establishing optimal protocols for APC preparation, dosing, and application, including inter-specialty consensus guidelines.

Biomaterial innovation focuses on the development of novel combination products (e.g., APCs with biomimetic scaffolds or synthetic sealants) that enhance both hemostatic and regenerative properties.

Patient-centered research, including studies on postoperative quality of life, pain reduction, and recovery timelines associated with different hemostatic strategies.

Such investigations will be key to optimizing clinical outcomes, ensuring cost-effective care, and broadening the applicability of advanced hemostatic technologies in oral surgery and beyond.

## Figures and Tables

**Table 1:** Factors influencing post-extraction bleeding and clinical relevance [11,12,13,14,15,16,17,18,19,20,21].

General Factors	Local Factors	Clinical Application/Recommendation Level
Uncontrolled hypertension	Pre-existing inflammation (e.g., periodontitis, infection) increases bleeding via vasodilation, vascular permeability, and impaired coagulation.	Common use–Control blood pressure preoperatively; manage inflammation to reduce bleeding.
Alcoholism and cirrhosis	Extent of surgical trauma (e.g., bone removal, tooth sectioning) raises vascular disruption.	Specialized use–Pre-op liver function assessment; minimize tissue trauma.
Diabetes	Soft tissue injury delays healing and increases bleeding risk.	Common use–Ensure glycemic control; apply atraumatic techniques.
Hemorrhagic diatheses (e.g., hemophilia, vasculitis, vitamin C/K deficiencies)	–	Specialized use–Requires hematology input; may need clotting factor replacement.
Hemorrhagic syndromes (e.g., leukemia, hepatitis, purpura)	–	Specialized use–Defer elective procedures if possible; use local and systemic strategies.
Anticoagulant/antiplatelet use (e.g., aspirin, warfarin, NOACs)	–	Common use–Usually do not discontinue; apply local agents (e.g., tranexamic acid, sutures).
Hormonal causes (e.g., menopause, metropathy)	–	Less common–Assess if unexplained bleeding; consider hormonal workup if persistent.

**Table 2:** Local hemostatic methods classification [11,12,13,14,15,16,17,18,19,20,21,22].

Local Hemostatic Methods	
Mechanical methods	Gauze compression
	Sutures
	Hemostatic clamps or clips
Chemical methods	Oxidized regenerated cellulose
	Gelatin sponges
	Collagen-based products
	Ferric sulfate or aluminum chloride
	Chitosan-based agents
Biological agents	Fibrin sealants
	Thrombin
	Platelet concentrates
Pharmacological Adjuncts	Vasoconstrictors in local anesthesia
	Antifibrinolytics

**Table 3:** Comparative analysis of autologous platelet concentrates (APC) with traditional hemostatic agents [4,11,17,23,24,25,26,27,28,29,30,31,32,33,34,35,36,37,38,39,40,41,42,43,44,45,46,47,48,49,50,51,52,53,54,55,56,57].

Parameter	APCs (PRP/PRF)	Traditional Hemostatic Agents
Healing Outcomes	Superior tissue regeneration and faster wound healing due to growth factors.	Effective for hemostasis, but limited regenerative potential.
Hemostatic Efficacy	Moderate to high (dependent on preparation quality and site).	High, especially with agents like thrombin, gelatin, or fibrin glue.
Infection Risk	Very low (autologous source).	Low, but higher than APCs due to foreign or animal-derived materials.
Cost	Higher initial cost due to equipment and processing.	Generally lower and more accessible.
Procedural Complexity	Requires blood draw, centrifugation, and preparation time (~10–20 min).	Simple and quick application (e.g., sponge, gauze, or solution).
Biocompatibility	Excellent (patient’s own blood).	Varies; some may cause reactions (e.g., bovine thrombin, gelatin).
Clinical Use Cases	Preferred in regenerative dentistry, implants, and periodontics.	Widely used in general and emergency hemostasis.

**Table 4:** Main characteristics of hemostatic agents.

Hemostatic Agent	Characteristics	Advantiges	Disadvantiges
Aprotinin [24,25]	Protease inhibitor; reduces fibrinolysis by inhibiting plasminogen activators.	Reduces post-operative bleeding; used to prevent fibrinolysis in high-risk surgeries.	Risk of allergic reactions; potential for kidney toxicity with prolonged use.
Etamsylate [27,28]	Hemostatic agent that stabilizes capillary walls and enhances platelet aggregation.	Reduces bleeding during and after surgery; can be used in conjunction with other hemostatic agents.	Limited effectiveness in major bleeding; not suitable as a standalone treatment in severe cases.
Topical Thrombin [42]	Directly converts fibrinogen to fibrin, aiding in clot formation.	Fast-acting; effective for controlling diffuse oozing; easy application as spray or gel.	May cause allergic reactions; derived from bovine sources, posing a risk of immunogenicity.
Aminocaproic Acid [18,29]	Antifibrinolytic; inhibits plasminogen activation.	Cost-effective; can be used both topically and systemically.	May increase the risk of thrombosis; less effective than other hemostatic agents in complex cases.
Epsylone (Aminocaproic Acid) [29]	Same as Aminocaproic Acid; commonly used for bleeding control in surgery.	Economical; easy to administer both orally and topically.	May cause side effects like nausea, diarrhea; contraindicated in patients with active thrombosis.
Paraaminomethylbenzoic Acid (PAMBA) [30,31]	A synthetic compound that enhances platelet aggregation and stabilizes fibrin.	Effective in enhancing clot formation; may improve outcomes in minor bleeding cases.	Not widely used in clinical practice; limited evidence on efficacy in oral surgery.
Alveo-Penga [40,41]	Combination of a hemostatic sponge and a polymer material designed for bleeding control.	Convenient and effective in oral surgeries; combines both hemostatic and wound healing properties.	Limited long-term data on its efficacy; may not be suitable for larger or more complex surgeries.
Gelaspon (Gelatin Sponge) [11,25]	Absorbable sponge derived from porcine or bovine gelatin.	Provides mechanical support for clot formation; absorbable; easy to handle.	Risk of infection if not absorbed; may cause foreign body reaction in rare cases.
Collagen Sponges [30]	Derived from bovine or porcine collagen; acts as a scaffold for platelet adhesion.	Biocompatible; promotes clot formation and wound healing; absorbable.	Cost may be high; limited hemostatic effect in active bleeding compared to other agents.
Oxidized Cellulose [22,32]	Plant-derived; creates an acidic environment that promotes clotting.	Effective for minor to moderate bleeding; bactericidal properties; absorbable.	May delay healing if remnants persist; ineffective in heavy bleeding or in the presence of heparin.
Tranexamic Acid [50]	Antifibrinolytic; inhibits plasminogen activation to prevent clot breakdown.	Effective for reducing bleeding; easy to use; can be applied topically or orally.	Risk of systemic side effects if absorbed; contraindicated in patients with thromboembolic disorders.
Fibrin Sealants [43,44]	Combines fibrinogen and thrombin to mimic the final stage of the clotting cascade.	Promotes both hemostasis and tissue healing; effective in complex surgical sites.	Expensive; risk of infection or immunological reaction due to human-derived components.
Autologous Platelet Concentrates (APCs) [45,51]	Derived from the patient’s blood; includes PRP (platelet-rich plasma) or L-PRF (leukocyte-platelet rich fibrin).	Combines hemostasis with tissue regeneration; minimizes risk of infection or immunological reactions.	Preparation can be time-consuming; requires additional equipment; effectiveness may vary depending on preparation quality.
PRP (Platelet-Rich Plasma) [52]	High concentration of platelets; releases growth factors for healing.	Enhances healing; reduces inflammation; patient-derived, minimizing rejection risks.	Requires processing time; expensive equipment; efficacy is highly technique-dependent.

## Data Availability

Not applicable.

## References

[1] Kathariya R., Devanoorkar A., Jain H. (2013). Intra-operative hemorrhage: A review of literature. J. Med. Diagn. Methods.

[2] Ahmed S. (2021). Management of Hemorrhage in Minor Dental Operations-A Systematic Review. Oral Maxillofac. Pathol. J..

[3] Kumar K.A., Kumar J., Sarvagna J., Gadde P., Chikkaboriah S. (2016). Hemostasis and post-operative care of oral surgical wounds by Hemcon dental dressing in patients on oral anticoagulant therapy: A split mouth randomized controlled clinical trial. J. Clin. Diagn. Res. JCDR.

[4] Ukaegbu K. (2024). Management of Bleeding in Dental Surgery: A Mini Review. SVOA Dent..

[5] Mani A., Anarthe R., Kale P., Maniyar S., Anuraga S., Student P.G. (2018). Hemostatic agents in dentistry. Galore Int. J. Health Sci. Res..

[6] Ghimire S., Sarkar P., Rigby K., Maan A., Mukherjee S., Crawford K.E., Mukhopadhyay K. (2021). Polymeric materials for hemostatic wound healing. Pharmaceutics.

[7] Mp S.K. (2016). Local hemostatic agents in the management of bleeding in oral surgery. Asian J. Pharm. Clin. Res..

[8] Irfan N.I., Zubir A.Z.M., Suwandi A., Haris M.S., Jaswir I., Lestari W. (2022). Gelatin-based hemostatic agents for medical and dental application at a glance: A narrative literature review. Saudi Dent. J..

[9] Ogle O.E., Swantek J., Kamoh A. (2011). Hemostatic agents. Dent. Clin..

[10] Scarano A., Murmura G., Di Cerbo A., Palmieri B., Pinchi V., Mavriqi L., Varvara G. (2013). Anti-hemorrhagic agents in oral and dental practice: An update. Int. J. Immunopathol. Pharmacol..

[11] Takizawa K., Okazaki D., Takegawa Y., Koga Y., Sagata M., Michishita K., Shinya N. (2020). Effectiveness of Local Hemostatic Agents in Oral Surgery for Patients on Anticoagulation Therapy. J. Oral Maxillofac. Surg..

[12] Andrade N.K.D., Motta R.H.L., Bergamaschi C.D.C., Oliveira L.B., Guimarães C.C., Araujo J.D.O., Lopes L.C. (2019). Bleeding risk in patients using oral anticoagulants undergoing surgical procedures in dentistry: A systematic review and me-ta-analysis. Front. Pharmacol..

[13] Inchingolo F., Inchingolo A.M., Piras F., Ferrante L., Mancini A., Palermo A., Dipalma G. (2024). Management of Patients Receiving Anticoagulation Therapy in Dental Practice: A Systematic Review. Healthcare.

[14] Peck M.T., Anderson J.M. (2021). Efficacy of Autologous Platelet Concentrates in Oral Surgery. J. Oral Surg. Res..

[15] Amaldhas J., Jimson S., Kannan I., Parthiban J. (2015). Assessment of bleeding during minor oral surgical procedures and extraction in patients on anticoagulant therapy. J. Pharm. Bioallied Sci..

[16] Zirk M., Fienitz T., Edel R., Kreppel M., Dreiseidler T., Rothamel D. (2016). Prevention of post-operative bleeding in hemostatic compromised patients using native porcine collagen fleeces—Retrospective study of a consecutive case series. Oral Maxillofac. Surg..

[17] Scottish Dental Clinical Effectiveness Programme (2015). Management of Dental Patients Taking Anticoagulants or Antiplatelet Drugs: Dental Clinical Guidance.

[18] Bajkin B.V., Bajkin I.A., Petrovic B.B. (2012). The effects of combined oral anticoagulant–aspirin therapy in patients undergoing tooth extractions. J. Am. Dent. Assoc..

[19] Blinder D., Manor Y., Martinowitz U., Taicher S. (1999). Dental extractions in patients maintained on continued oral anticoagulant: Comparison of local hemostatic modalities. Oral Surg. Oral Med. Oral Pathol. Oral Radiol. Endod..

[20] Gröbe A., Fraederich M., Smeets R., Heiland M., Kluwe L., Zeuch J., Haase M., Wikner J., Hanken H., Semmusch J. (2015). Postoperative Bleeding Risk for Oral Surgery under Continued Clopidogrel Antiplatelet Therapy. BioMed Res. Int..

[21] Hanken H., Tieck F., Kluwe L., Smeets R., Heiland M., Precht C., Eichhorn M., Eichhorn W. (2015). Lack of evidence for increased postoperative bleeding risk for dental osteotomy with continued aspirin therapy. Oral Surg. Oral Med. Oral Pathol. Oral Radiol..

[22] Morimoto Y., Niwa H., Minematsu K. (2008). Hemostatic management of tooth extractions in patients on oral antithrombotic therapy. J. Oral Maxillofac. Surg..

[23] Zhu G., Wang Q., Lu S., Niu Y. (2017). Hydrogen Peroxide: A Potential Wound Therapeutic Target. Med. Princ. Pract..

[24] Schneeweiss S., Seeger J.D., Landon J., Walker A.M. (2008). Aprotinin during Coronary-Artery Bypass Grafting and Risk of Death. N. Engl. J. Med..

[25] Lyseng-Williamson K.A. (2019). Aprotinin in adults at high risk of major blood loss during isolated CABG with cardiopulmonary bypass: A profile of its use in the EU. Drugs Ther. Perspect..

[26] Lee T., Huang C., Chang P., Chang C., Chen Y. (2009). Hemostasis during functional endoscopic sinus surgery: The effect of local infiltration with adrenaline. Otolaryngol. Neck Surg..

[27] Negrete O.R., Molina M., Gutierrez-Aceves J. (2009). Preoperative administration of ethamsylate: Reduces blood loss associated with percutaneous nephrolithotomy? A prospective randomized study. J. Urol..

[28] Albiñana V., Giménez-Gallego G., García-Mato A., Palacios P., Recio-Poveda L., Cuesta A.-M., Patier J.-L., Botella L.-M. (2019). Topically Applied Etamsylate: A New Orphan Drug for HHT-Derived Epistaxis (Antiangiogenesis through FGF Pathway Inhibition). TH Open.

[29] Levine M., Huang M., Henderson S.O., Carmelli G., Thomas S.H. (2016). Aminocaproic Acid and Tranexamic Acid Fail to Reverse Dabigatran-Induced Coagulopathy. Am. J. Ther..

[30] Steinmetzer T., Pilgram O., Wenzel B.M., Wiedemeyer S.J.A. (2019). Fibrinolysis Inhibitors: Potential Drugs for the Treatment and Prevention of Bleeding. J. Med. Chem..

[31] Estcourt L.J., Desborough M., Brunskill S.J., Doree C., Hopewell S., Murphy M.F., Stanworth S.J. (2016). Antifibrinolytics (lysine analogues) for the prevention of bleeding in people with haematological disorders. Cochrane Database Syst. Rev..

[32] Halfpenny W., Fraser J.S., Adlam D.M. (2001). Comparison of 2 hemostatic agents for the prevention of postextraction hemorrhage in patients on anticoagulants. Oral Surg. Oral Med. Oral Pathol. Oral Radiol. Endodontol..

[33] Mladenov D.A., Tsvetkov T.D., Vulchanov N.L. (1993). Freeze Drying of Biomaterials for the Medical Practice. Cryobiology.

[34] Speechley J.A. (2008). Dry socket secrets. Br. Dent. J..

[35] Lew W.K., Weaver F.A. (2008). Clinical use of topical thrombin as a surgical hemostat. Biol. Targets Ther..

[36] Lozano A.C., Perez M.S., Esteve C.G. (2011). Dental management in patients with hemostasis alteration. J. Clin. Exp. Dent..

[37] Mankad P.S., Codispoti M. (2001). The role of fibrin sealants in haemostasis. Am. J. Surg..

[38] Mahmoudi A., Ghavimi M.A., Dizaj S.M., Sharifi S., Sajjadi S.S., Khosroshahi A.R.J. (2023). Efficacy of a New Hemostatic Dental Sponge in Controlling Bleeding, Pain, and Dry Socket Following Mandibular Posterior Teeth Extraction—A Split-Mouth Randomized Double-Blind Clinical Trial. J. Clin. Med..

[39] Lillis T., Ziakas A., Koskinas K., Tsirlis A., Giannoglou G. (2011). Safety of Dental Extractions During Uninterrupted Single or Dual Antiplatelet Treatment. Am. J. Cardiol..

[40] Spotnitz W.D. (2014). Fibrin Sealant: The Only Approved Hemostat, Sealant, and Adhesive—A Laboratory and Clinical Perspective. ISRN Surg..

[41] Martinowitz U., Mazar A.L., Taicher S., Varon D., Gitel S.N., Ramot B., Rakocz M. (1990). Dental extraction for patients on oral anticoagulant therapy. Oral Surg. Oral Med. Oral Pathol..

[42] Cardona-Tortajada F., Sainz-Gomez E., Figuerido-Garmendia J., de Robles-Adsuar A.L., Morte-Casabo A., Giner-Munoz F., Artazcoz-Oses J., Vidan-Lizari J. (2009). Dental extractions in patients on antiplatelet therapy. A study conducted by the Oral Health Department of the Navarre Health Service. Med. Oral Patol. Oral Cir. Bucal.

[43] Carter G., Goss A., Lloyd J., Tocchetti R. (2003). Tranexamic acid mouthwash versus autologous fibrin glue in patients taking warfarin undergoing dental extractions: A randomized prospective clinical study. J. Oral Maxillofac. Surg..

[44] Perry D.J., Noakes T.J.C., Helliwell P.S. (2007). Guidelines for the management of patients on oral anticoagulants requiring dental surgery. Br. Dent. J..

[45] Eberhard U., Broder M., Witzke G. (2006). Stability of Beriplast P fibrin sealant: Storage and reconstitution. Int. J. Pharm..

[46] Fortelny R.H., Petter-Puchner A.H., Glaser K.S., Redl H. (2012). Use of fibrin sealant (Tisseel/Tissucol) in hernia repair: A systematic review. Surg. Endosc..

[47] Al-Mubarak S., Al-Ali N., Rass M.A., Al-Sohail A., Robert A., Al-Zoman K., Al-Suwyed A., Ciancio S. (2007). Evaluation of dental extractions, suturing and INR on postoperative bleeding of patients maintained on oral anticoagulant therapy. Br. Dent. J..

[48] Kamal S.M., Hegab D.S., El Maghraby G.M., Ashmawy A.A.E. (2023). Efficacy and Safety of Topical Tranexamic Acid Alone or in Combination with Either Fractional Carbon Dioxide Laser or Microneedling for the Treatment of Melasma. Dermatol. Pract. Concept..

[49] Hupp J.R., Ellis E., Tucker M.R. (2014). Contemporary Oral and Maxillofacial Surgery.

[50] Mani M., Ebenezer V., Balakrishnan R. (2014). Impact of Hemostatic Agents in Oral Surgery. Biomed. Pharmacol. J..

[51] Chmielewski M., Pilloni A., Adamska P. (2024). Application of Advanced Platelet-Rich Fibrin in Oral and Maxillo-Facial Surgery: A Systematic Review. J. Funct. Biomater..

[52] Shaikh Z.S., Thompson A.R., Halsnad M., Fowell C.J., Millar B.G., Grew N.R., Pigadas N. (2011). The clinical applications of platelet-rich plasma in oral and maxillofacial surgery. Br. J. Oral Maxillofac. Surg..

[53] Ugale G.M., Male R.B., Bhandari V.D., Baghele O.N., Metri R., Ugale M.S. (2023). Evaluation of platelet-rich fibrin in the treatment of decorticated intrabony defects: A randomized clinical trial. Quintessence Int..

[54] Marenzi G., Riccitiello F., Tia M., di Lauro A., Sammartino G. (2015). Influence of Leukocyte- and Platelet-Rich Fibrin (L-PRF) in the Healing of Simple Postextraction Sockets: A Split-Mouth Study. BioMed Res. Int..

[55] Roberts G., Scully C., Shotts R. (2000). ABC of oral health: Dental emergencies. BMJ.

[56] Strauss E.R., Taneja M., Booth R., Sankova S., Anders M.G. (2022). Antifibrinolytics in Cardiac Surgery: What Is the Best Practice in 2022?. Curr. Anesthesiol. Rep..

[57] Aldhaeefi M., Badreldin H.A., Alsuwayyid F., Alqahtani T., Alshaya O., Al Yami M.S., Bin Saleh K., Al Harbi S.A., Alshaya A.I. (2023). Practical Guide for Anticoagulant and Antiplatelet Reversal in Clinical Practice. Pharmacy.

[58] Borle R.M. (2014). Textbook of Oral and Maxillofacial Surgery.

[59] Faris A., Khalid L., Hashim M., Yaghi S., Magde T., Bouresly W., Hamdoon Z., Uthman A.T., Marei H., Al-Rawi N. (2022). Characteristics of Suture Materials Used in Oral Surgery: Systematic Review. Int. Dent. J..

[60] Koshak H.H. (2017). Dental Suturing Materials and Techniques. Glob. J. Otolaryngol..

[61] Dinkova A. (2024). Hemostasis and Surgical Treatment in Dental Medicine.

[62] Szumita R.P., Szumita P.M., Szumita R., Szumita P. (2018). Local Techniques and Pharmacologic Agents for Management of Bleeding in Dentistry. Haemostasis in Dentistry.

